# *Helicobacter pylori* and Impaired Early Childhood Development—Evidence From a Birth Cohort Study From Ghana and Côte d'Ivoire

**DOI:** 10.1111/hel.70087

**Published:** 2025-10-31

**Authors:** Kirsten Alexandra Eberhardt, Stefanie Schoppen, Carola Bindt, Stephan Ehrhardt, Yasmin Mohammed, Daniel Fordjour, Doris Kra‐Yao, Carine Esther Bony Kotchi, Ekissi Jean Koffi Armel, Bernardin Ahouty Ahouty, Rebecca Hinz, John Appiah‐Poku, Mathurin Koffi, Samuel Blay Nguah, Harry Tagbor, Eliezer N'Goran, Richard Odame Phillips, Tom Luedde, Dana Barthel, Torsten Feldt

**Affiliations:** ^1^ Department of Gastroenterology, Hepatology and Infectious Diseases, Medical Faculty and University Hospital Düsseldorf Heinrich Heine University Düsseldorf Düsseldorf Germany; ^2^ Department of Tropical Medicine Bernhard Nocht Institute for Tropical Medicine Hamburg Germany; ^3^ Department of Health and Social Science Hochschule Fresenius Hamburg Germany; ^4^ Department of Child and Adolescent Psychiatry, Psychotherapy, and Psychosomatics University Medical Center Hamburg‐Eppendorf Hamburg Germany; ^5^ Department of Epidemiology Johns Hopkins Bloomberg School of Public Health Baltimore Maryland USA; ^6^ Infectious Diseases Centre, Department of Medicine and Therapeutics Korle Bu Teaching Hospital Accra Ghana; ^7^ Department of Physician Assistantship Studies, School of Health and Allied Sciences Garden City University College Kumasi Ghana; ^8^ Laboratoire de Santé, Nutrition et Hygiène, Centre de Recherche Pour le Développement Université Alassane Ouattara Bouaké Côte d'Ivoire; ^9^ Département de Psychologie Université Félix Houphouët‐Boigny de Cocody Abidjan Côte d'Ivoire; ^10^ Université Jean Lorougnon Guédé, Unité de Recherche en Génétique et Épidémiologie Moléculaire UFR Environnement Daloa Côte d'Ivoire; ^11^ Department of Clinical Microbiology Labor Dr. Heidrich & Kollegen Hamburg Germany; ^12^ Department of Behavioural Sciences, School of Medicine and Dentistry Kwame Nkrumah University of Science and Technology Kumasi Ghana; ^13^ Department of Child Health, School of Medicine and Dentistry Kwame Nkrumah University of Science and Technology Kumasi Ghana; ^14^ Directorate of Child Health Komfo Anokye Teaching Hospital Kumasi Ghana; ^15^ Department of Community Health, School of Medicine University of Health and Allied Sciences Ho Ghana; ^16^ Unité de Formation et de Recherche Biosciences Université Félix Houphouët‐Boigny Abidjan Côte d'Ivoire; ^17^ Kumasi Centre for Collaborative Research in Tropical Medicine Kwame Nkrumah University of Science and Technology Kumasi Ghana; ^18^ Department of Medicine, School of Medical Sciences Kwame Nkrumah University of Science and Technology Kumasi Ghana

**Keywords:** *H. pylori*, infection control, motor development, neurodevelopment, quantile regression, sub‐Saharan Africa

## Abstract

**Background:**

*Helicobacter pylori* ( *H. pylori* ) infection is highly prevalent in low‐resource settings and has been implicated in adverse health outcomes beyond the gastrointestinal tract, including potential effects on early neurodevelopment. However, data from sub‐Saharan Africa is limited.

**Methods:**

We conducted a prospective cohort study among 229 mother–child dyads from Ghana and Côte d'Ivoire to assess the association between *H. pylori* infection and early child development at 12 months of age. Child development was evaluated using the Developmental Milestones Checklist (DMC), encompassing locomotor, fine motor, language, and personal–social domains. *H. pylori* infection status was determined by stool antigen testing. Quantile regression models, adjusted for socioeconomic status and sex, were applied to analyze associations between *H. pylori* infection and DMC scores.

**Results:**

Among 229 children, 38 (16.6%) tested positive for *H. pylori* . The median total motor score was higher in *H. pylori* ‐negative children (45, IQR 41–50) compared to positive children (43, IQR 40–45; *p* = 0.031). At the 75th percentile, *H. pylori* infection was significantly associated with a 7‐point reduction in the motor domain score (estimate = −7; 95% CI: −11.1 to −2.9; *p* = 0.001; FDR‐adjusted *p* = 0.003) and a 10‐point reduction in total DMC score (estimate = −10; 95% CI: −17.5 to −2.6; *p* = 0.009; FDR‐adjusted *p* = 0.027) after multivariable adjustment. No significant associations were observed at lower or median quantiles, nor in language or personal‐social domains.

**Conclusion:**

*H. pylori* infection at 12 months of age is significantly associated with impaired motor development among children performing in the upper range of developmental achievement. These findings underscore the potential neurodevelopmental impact of early‐life *H. pylori* infection in high‐prevalence, resource‐limited settings and highlight the importance of integrating infection control strategies into early childhood development programs. Further research is warranted to elucidate infection‐related neurodevelopmental risks in early childhood.

## 1 Introduction

*Helicobacter pylori* ( *H. pylori* ) is a Gram‐negative, microaerophilic bacterium that colonizes the gastric mucosa and is among the most common chronic infections globally [1, 2]. Recent meta‐analyses estimate its worldwide prevalence at approximately 44%–50%, though rates vary markedly by region and socioeconomic status [3, 4]. Adult seroprevalence of *H. pylori* in high‐income countries typically ranges from 20% to 40%, whereas in resource‐limited settings, especially in sub‐Saharan Africa, rates often exceed 70% [5, 6, 7]. In these high‐income regions, childhood prevalence is relatively low and acquisition occurs gradually over time, while in low‐resource settings, such as West Africa, *H. pylori* colonization often begins within the first years of life, with seroprevalence reaching 60% to 80% by the age of five [8, 9]. Early childhood acquisition is reported to be associated with overcrowding, poor sanitation, and limited access to healthcare [10, 11]. In West Africa, particularly in Ghana, the burden of *H. pylori* infection remains substantial. A recent cross‐sectional study conducted among Hepatitis B infected individuals in the Greater Accra Region reported an *H. pylori* prevalence of 41% [12]. High prevalences have also been reported among adult Ghanaians with dyspepsia (around 75%) and in rural children (approximately 14%) [10, 13]. While prevalence data for Côte d'Ivoire are more limited, available studies and regional reviews report high rates of *H. pylori* infection exceeding 66% and reaching up to 92% among symptomatic patients [14, 15].

Chronic *H. pylori* infection is a well‐established risk factor for peptic ulcer disease, gastric adenocarcinoma, and mucosa‐associated lymphoid tissue (MALT) lymphoma [7, 16]. Additionally, evidence links *H. pylori* to extragastric conditions, including iron deficiency anemia and idiopathic thrombocytopenic purpura [17]. In pediatric populations, while overt gastrointestinal disease is rare, infection has been associated with growth faltering, micronutrient deficiencies, and systemic inflammation [11, 18, 19]. These observations raise concerns that chronic infection during critical periods of development may have broader health impacts beyond the gastrointestinal tract.

Child development, encompassing motor, cognitive, language, and socio‐emotional domains, is particularly sensitive in the first years of life [20]. Validated screening tools, such as the Bayley Scales of Infant Development and the Developmental Milestones Checklist, are available to assess milestones in one‐year‐old children, and their use is essential for early identification of developmental delays, which strongly predict later educational and health outcomes [21, 22, 23, 24, 25].

Emerging evidence suggests a potential link between *H. pylori* infection and neurodevelopmental outcomes. In adults, several studies have associated *H. pylori* seropositivity with cognitive impairment and dementia, possibly mediated by micronutrient malabsorption and chronic inflammation [26, 27, 28]. Although direct evidence in children remains limited, a recent cohort study reported lower neurodevelopmental scores in young children with *H. pylori* infection, suggesting that early‐life exposure may adversely affect brain development [29]. Most research to date has been conducted in high‐income countries, while data from low‐ and middle‐income regions, where both infection prevalence and risk factors for developmental delay are high, remain scarce [25, 30, 31].

Addressing this gap, the present study investigates the association between *H. pylori* positivity and early motor development in one‐year‐old children from a West African birth cohort. By focusing on a high‐prevalence, resource‐limited setting, this research aims to contribute valuable data to an underexplored area of global child health. Unimpaired neurocognitive development is a foundational determinant of a population's educational attainment and, by extension, its broader regional developmental and socioeconomic trajectory.

## 2 Methods

### 2.1 Study Design and Setting

**FIGURE 1:**
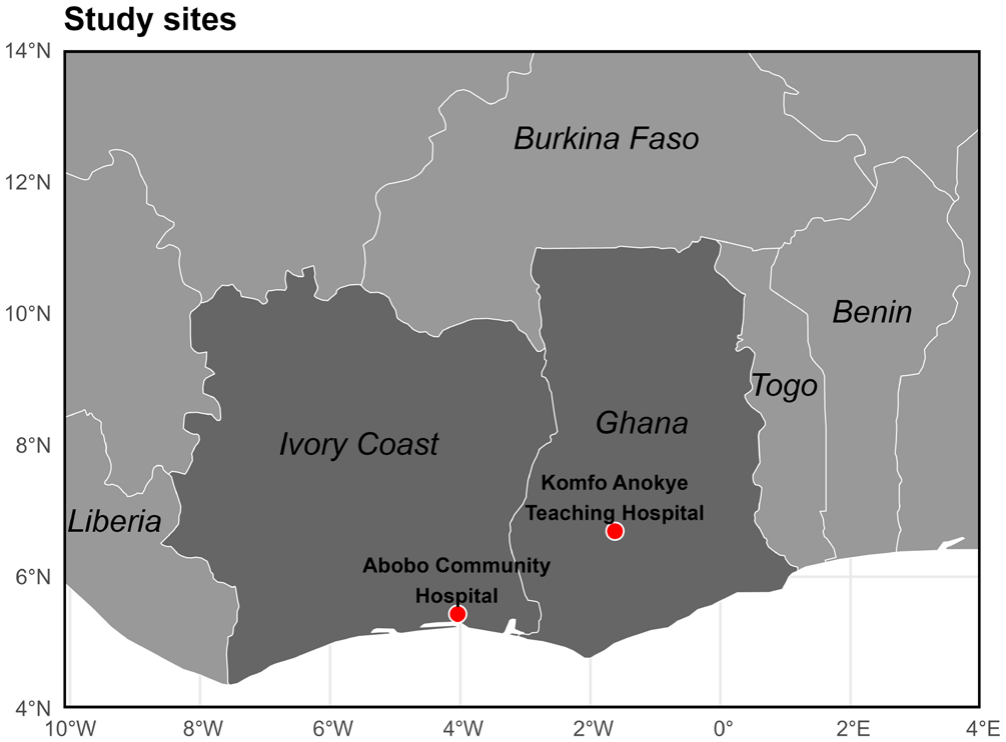
Geographic locations of study sites in West Africa. Geographic map of West Africa indicating the locations of the two study sites: Komfo Anokye Teaching Hospital in Kumasi, Ghana, and Abobo Community Hospital in Abobo, Côte d'Ivoire, represented by red dots. The map was created using R (version 4.4.3) employing the rnaturalearth package for accurate country boundaries and geographic data.

This study is part of the Child Development Study (CDS), a prospective birth cohort study of women and their children in Ghana and Côte d'Ivoire [32, 33]. Between March 2010 and December 2011, pregnant women in their third trimester were consecutively recruited during antenatal visits at two hospitals: Komfo Anokye Teaching Hospital in Kumasi, Ghana, serving a general urban population, and Abobo Community Hospital in Abidjan, Côte d'Ivoire, which provides care to a socioeconomically disadvantaged community affected by civil conflicts during that period (Figure 1).

Kumasi, Ghana's second‐largest city with an estimated population between 1.6 and 3 million, faces urbanization challenges including high population density and variable access to sanitation infrastructure. Although open defecation is largely eliminated, inconsistent drainage and overcrowding sustain risks for infection transmission [34]. Abobo is a low‐income, densely populated district of Abidjan, Côte d'Ivoire, where rapid urban migration and informal settlement growth following civil conflicts (2002–2011) have strained public services and sanitation infrastructure [35]. Limited access to improved sanitation increases vulnerability to waterborne and fecal‐oral infections, with close living quarters and communal spaces further facilitating transmission [35].

Mothers with high obstetric risk including multiple pregnancy, and HELLP syndrome or pre‐eclampsia, as well as children with severe birth complications such as prematurity, low Apgar scores, low birth weight, severe jaundice, or birth hemorrhage requiring transfusion, were excluded to minimize confounding. The current study includes data from the assessments that took place at recruitment (approximately 3 months before expected birth), at birth and at 12 months follow‐up assessments after birth. The study was approved by the ethical committees of the Kwame Nkrumah University of Science and Technology in Kumasi, Ghana, the National Ethical Committee in Abidjan, Côte d'Ivoire, and the Chamber of Physicians in Hamburg, Germany. All participating mothers provided written informed consent.

### 2.2 Clinical and Sociodemographic Data

A range of sociodemographic factors collected during antenatal recruitment, along with selected obstetric indicators recorded at birth, were documented for analysis. A socioeconomic status (SES) index was derived using principal component analysis (PCA) based on six indicators: household ownership of a refrigerator, car, and bednet, type of toilet facility, and the educational attainment of both the mother and her spouse. The median was used to differentiate between low and high SES.

### 2.3 Assessment of Early Child Development

The Developmental Milestones Checklist (DMC) is a structured tool for assessing early childhood development in the following domains: locomotor, fine motor, language, and personal‐social skills, typically between 3 and 24 months of age and has been validated for use in the Sub‐Saharan African context [36, 37]. Unlike more commonly used tools in high‐income settings, the DMC uses items that have been culturally adapted and specifically validated to ensure relevance and accuracy for this population. It uses caregiver‐directed questions and brief observation to identify children at risk for developmental delays (Figure S1).

### 2.4 Laboratory Analysis

Native stool samples were collected from mothers at the timepoint of recruitment and children with 12 months of age. Samples were freshly frozen at −80°C and transported to Germany on dry ice. Stool was tested for *H. pylori* using the RidaScreen FemtoLab *H. pylori* stool antigen test (R‐Biopharm AG, Germany). The sensitivity and specificity of this test have been described to be 98% and 96.7% in pediatric patients [38]. This test detects active infection by identifying bacterial antigen in stool, offering an accurate assessment of current *H. pylori* colonization. Additionally, a blood sample was obtained from each child for routine laboratory analysis, to assess general health and detect possible anemia or infection.

### 2.5 Statistical Analysis

Descriptive analyses of the *H. pylori* positive and negative study populations were conducted. Categorical variables were compared using the Fisher exact test. Continuous variables were assessed for normality using the Shapiro–Wilk test; non‐normally distributed parameters were summarized as median (IQR) and compared with the Wilcoxon rank sum test. To analyze the association between *H. pylori* status, potential confounders, and the right‐skewed psychometric outcome, univariable and multivariable quantile regression (QR) models were used. Unlike linear regression, which estimates effects on the conditional mean of the outcome, quantile regression models the effects of predictors across different points of the outcome distribution, offering greater robustness to outliers and skewed data and providing a more comprehensive understanding of the relationship between *H. pylori* infection and DMC scores [39, 40]. Specifically, the models estimate associations at the 25th, 50th, and 75th percentiles, corresponding to children with lower, median, and higher developmental performance, respectively, allowing for the characterization of the differential impact of *H. pylori* infection across the distribution of developmental outcomes. For each domain‐specific model, false discovery rate (FDR) correction was applied separately to the *p*‐values associated with *H. pylori* as the primary exposure. Statistical analyses were conducted using the software R (version 4.4.3, R Foundation for Statistical Computing, Vienna, Austria). All statistical tests were two‐sided, and a *p*‐value of < 0.05 was considered statistically significant.

## 3 Results

### 3.1 Study Population

A total of 1030 pregnant women (299 from Ghana, 731 from Côte d'Ivoire) were recruited in their third trimester for the original CDS study. More participants were enrolled in Côte d'Ivoire due to losses of data, biological samples, or contact with study participants during an armed conflict and subsequent additional recruitment. Following the exclusion of women who either delivered outside the study hospitals or were lost to follow‐up, largely due to the armed conflict, 264 mother–child dyads completed the 12‐month follow‐up assessment [41]. Of these, stool samples from 229 (87%) one‐year‐old infants were available for *H. pylori* testing.

### 3.2 Demographic, Socioeconomic, and Clinical Characteristics by *H. pylori* Status

Table 1 presents a descriptive analysis of demographic, socioeconomic, anthropometric, and laboratory parameters between children who tested negative and those who tested positive for *H. pylori* at 12 months of age. The sample consists of 229 children, with 191 (83.4%) classified as *H. pylori* ‐negative and 38 (16.6%) as *H. pylori* ‐positive. Sex distribution was similar between groups, with males comprising 54.5% of the negative group and 47.4% of the positive group. Country and socioeconomic status also did not differ significantly, with children almost evenly split between Côte d'Ivoire and Ghana and between high and low socioeconomic backgrounds in both groups. The median age at assessment and the timing of sample collection relative to the 12‐month mark were similar between groups. Anthropometric measures such as length and weight showed no significant differences, with median lengths of 75 and 74 cm, and median weights of 8900 and 8700 g, respectively. Breastfeeding as the main source of nutrition was reported in 46.8% of the negative group and 36.8% of the positive group (*p* = 0.288). Apgar scores at 1 min were identical, and the age and number of previous pregnancies among mothers was also similar. A notable difference emerged in the maternal *H. pylori* status at the time of recruitment. Among *H. pylori* ‐positive children, 91.4% had mothers who were *H. pylori* ‐positive, compared to 74.1% in the negative group (*p* = 0.027). Most laboratory values, including white blood cell count, hemoglobin, hematocrit, and mean corpuscular volume, were similar between groups. The red blood cell (RBC) count was slightly higher in the *H. pylori* ‐positive group (median 4.7 vs. 4.5, *p* = 0.026).

**TABLE 1:** Demographic, socioeconomic, anthropometric, and laboratory parameters in children at 12 months of age according to *H. pylori* status.

	Variable	*H. pylori* negative, *n* = 191 (83.4%)	*H. pylori* positive, *n* = 38 (16.6%)	*p*
Demographic and socioeconomic characteristics	Sex			0.478
	Male, *n* (%)	104 (54.45%)	18 (47.37%)	
	Female, *n* (%)	87 (45.55%)	20 (52.63%)	
	Country			0.377
	Côte d'Ivoire, *n* (%)	94 (49.21%)	22 (57.89%)	
	Ghana, *n* (%)	97 (50.79%)	16 (42.11%)	
	Socioeconomic status			0.479
	Low, *n* (%)	102 (54.26%)	18 (47.37%)	
	High, *n* (%)	86 (45.74%)	20 (52.63%)	
Perinatal and growth parameters	Date difference to 12 months in days (IQR)	4 (2/12)	6 (2/13)	0.490
	Length in cm (IQR)	75 (72/76)	74 (72/76)	0.704
	Weight in g (IQR)	8900 (8200/9633)	8700 (8383/9754)	0.730
	Breastfeeding as the main source of nutrition, *n* (%)	87 (46.77%)	14 (36.84%)	0.288
	Apgar score 1 min (IQR)	8 (7/8)	8 (7/8)	0.932
Maternal parameters	Age in years (IQR)	29 (25/34)	28 (25/34)	0.690
	Maternal *H. pylori* status			0.027
	Negative, *n* (%)	45 (25.86%)	3 (8.57%)	
	Positive, *n* (%)	129 (74.14%)	32 (91.43%)	
	Number of previous pregnancies, (IQR)	3 (1/4)	3 (1/4)	0.837
Laboratory Parameters	White blood cell (WBC), (IQR)	10 (8/13)	10 (9/13)	0.311
	Red blood cell (RBC) Count, (IQR)	4.5 (4.2/4.8)	4.7 (4.4/5.1)	0.026
	Hemoglobin (Hb), (IQR)	10 (9/11)	10 (10/11)	0.421
	Hematocrit (Hct), (IQR)	32 (29/34)	32 (28/34)	0.799
	Mean corpuscular volume (MCV), (IQR)	68 (62/74)	66 (62/72)	0.321

Abbreviation: IQR, interquartile range.

### 3.3 Association of *H. pylori* Infection With Early Child Development

Table 2 provides a comparative overview of DMC scores at 12 months of age between children who tested negative and those who tested positive for *H. pylori* . Four domains of the psychometric assessment are presented: locomotor, fine motor, language, and personal‐social. A total motor score and total score of all domains are presented as well. For this analysis, data of 184 *H. pylori* ‐negative children (82.9%) and 38 *H. pylori* ‐positive children (17.1%) were available. The median motor score was slightly higher in the *H. pylori* ‐negative group (45, IQR 41–50) compared to the positive group (43, IQR 40–45), with a *p*‐value of 0.031. In contrast to this, median language scores were similar between groups: 13 (IQR 9–19) for *H. pylori* ‐negative and 14 (IQR 9–19) for *H. pylori* ‐positive children. Personal‐social scores were also nearly identical: 32 (IQR 30–35) in the negative group and 32 (IQR 31–34) in the positive group. The total DMC score, reflecting overall developmental status, was slightly higher in the *H. pylori* ‐negative group (median 92, IQR 82–108) than in the positive group (median 89, IQR 84–97), although this difference did not reach statistical significance (*p* = 0.359).

**TABLE 2:** Developmental milestone checklist (DMC) scores at 12 months of age according to *H. pylori* status.

	*H. pylori* negative, *n* = 184 (82.9%)	*H. pylori* positive, *n* = 38 (17.1%)	*p*
Locomotor domain (IQR)	26 (24/30)	25 (23/28)	0.262
Fine motor domain (IQR)	17 (16/19)	17 (16/18)	0.086
Language domain (IQR)	13 (9/19)	14 (9/19)	0.697
Personal‐social domain (IQR)	32 (30/35)	32 (31/34)	0.443
Total motor score (IQR)	45 (41/50)	43 (40/45)	0.031
Total score (IQR)	92 (82/108)	89 (84/97)	0.359

Abbreviation: IQR, interquartile range.

**FIGURE 2:**
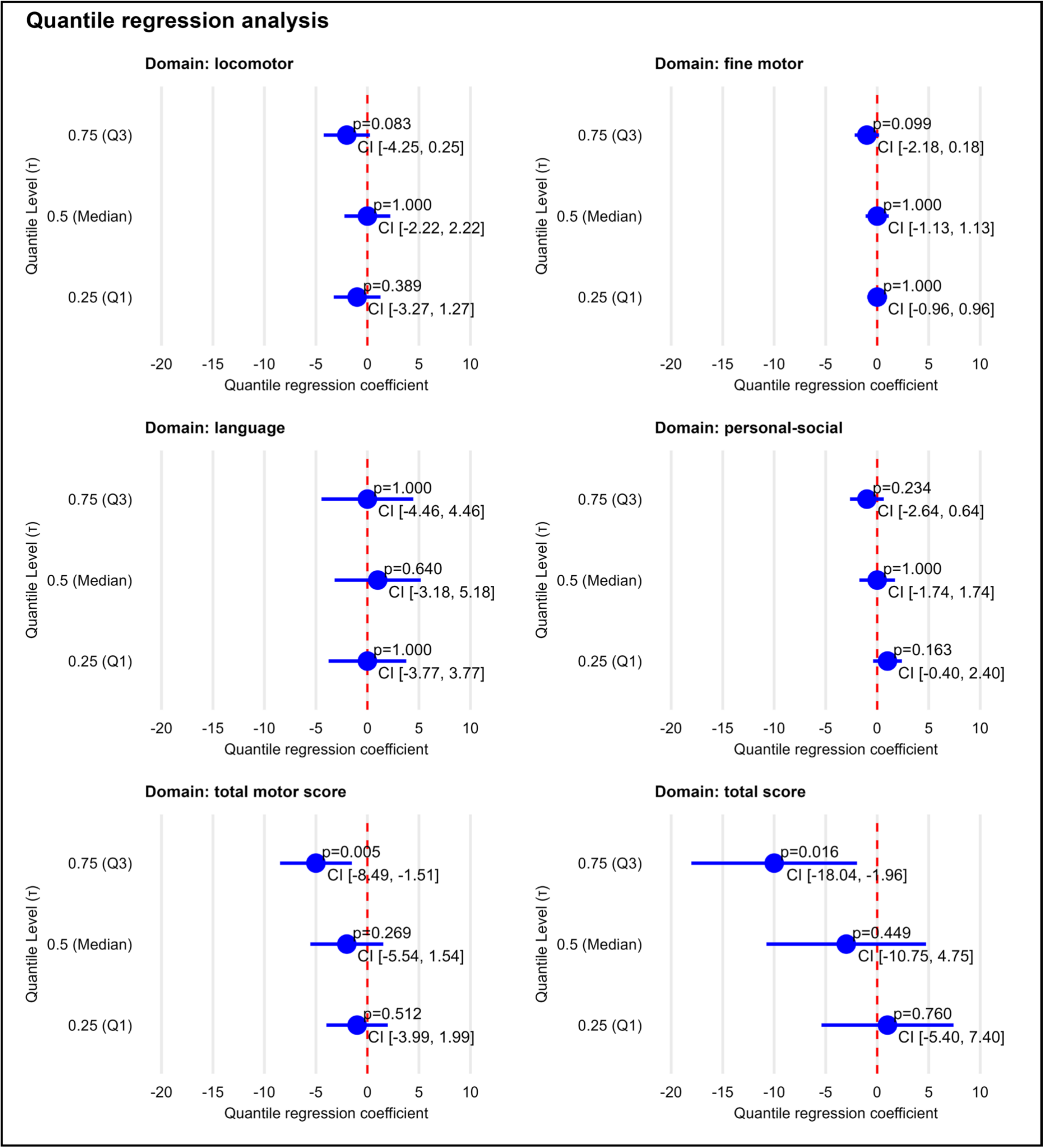
Association between *H. pylori* status and early child developmental outcomes. Results from univariate quantile regression analysis showing associations between *H. pylori* status and developmental milestone scores in 12‐month‐old children at the 25th percentile, median and 75th percentile. CI, confidence Interval; T, Tau (Quantile Level).

Figure 2 and Table S1 present the results of a univariable quantile regression analysis investigating the association between *H. pylori* infection and DMC scores at 12 months of age. The analysis examines three points in the distribution of the outcome scores: the 25th percentile (*τ* = 0.25), the median (*τ* = 0.5), and the 75th percentile (*τ* = 0.75). For each quantile, the estimated difference in the domain‐specific score associated with *H. pylori* positivity is reported alongside 95% confidence intervals. For the total motor development score, which sums up result scores from the locomotion and fine motor domains, no significant association was observed at the 25th percentile or the median. However, at the 75th percentile, *H. pylori* infection was significantly associated with a five‐point reduction in motor scores (estimate = −5; 95% CI: −8.5 to −1.5; *p* = 0.005; FDR‐adjusted *p* = 0.015). Regarding language and personal‐social development scores, the quantile regression analysis found no significant associations with *H. pylori* infection at any of the examined quantiles. When considering the total DMC score, the analysis again revealed no significant association at the lower and median quantiles. In contrast, at the 75th percentile, *H. pylori* positivity was linked to a significant 10‐point decrease in total developmental scores (estimate = −10; 95% CI: −18.0 to −2.0; *p* = 0.016; FDR‐adjusted *p* = 0.048).

Importantly, these associations persisted even after adjusting for potential confounding variables such as socioeconomic status and sex [42]. In the adjusted multivariable quantile regression model, *H. pylori* infection remained significantly associated with lower scores at the 75th percentile for the fine motor domain, the total motor score (estimate = −2; 95% CI: −3.3 to −0.7; *p* = 0.002; FDR‐adjusted *p* = 0.006 and estimate = −7; 95% CI: −11.1 to −2.9; *p* = 0.001; FDR‐adjusted *p* = 0.003, respectively) and total outcome score (estimate = −10; 95% CI: −17.5 to −2.6; *p* = 0.009; FDR‐adjusted *p* = 0.027). Specifically, the negative linkage of *H. pylori* on children performing in the upper range of developmental scores was robust to adjustment (Table S2). In contrast to the *H. pylori* status in children, *H. pylori* positivity in mothers was not found to be associated with child development in any quantile of the tested domains and total scores of the psychometric screening tool (Table S3).

## 4 Discussion

In this prospective cohort study of mother–child dyads from Côte d'Ivoire and Ghana, we found that *H. pylori* infection at 12 months of age was significantly associated with lower developmental milestone scores at the 75th percentile, particularly affecting motor skills. These associations persisted after adjusting for potential confounders such as socioeconomic status and child sex [42]. Given that gross and fine motor milestones follow well‐defined developmental trajectories and are easily observable, their assessment at 12 months is considered particularly reliable [43]. While the Developmental Milestones Checklist also captures language and social–emotional skills, assessment of these domains at this age often reflects the natural variability in their emergence and incorporates caregiver‐reported information [44, 45]. Both delays and advanced attainment of early motor milestones have been linked to later cognitive and adaptive outcomes, with early motor skills initiating developmental cascades across cognitive, language, and social domains [46]. No significant associations were observed at the lower or median quantiles, and maternal *H. pylori* serostatus was not predictive of child developmental outcomes.

Our findings are consistent with emerging literature suggesting a link between *H. pylori* infection and neurodevelopmental outcomes in children. For example, a study in Israel found that *H. pylori* infection was associated with lower cognitive scores in school‐aged children, independent of socioeconomic and nutritional factors [47]. Another study from the Rhea birth cohort in Greece similarly reported associations between *H. pylori* seropositivity and poorer cognitive outcomes in early childhood [29]. While most research has focused on adults, where *H. pylori* infection has been linked to cognitive impairment via chronic inflammation and micronutrient deficiencies, evidence in pediatric populations remains limited and is often derived from high‐income settings with lower infection prevalence and different environmental exposures [48]. To our knowledge, our study is the first to investigate this association in sub‐Saharan Africa, a region characterized by early and widespread *H. pylori* acquisition and multiple risk factors for developmental delay.

Several mechanisms may explain the observed association between *H. pylori* infection and impaired neurodevelopment. Chronic gastric inflammation in infancy can impair nutrient absorption during critical periods of brain development. Deficiencies in iron, vitamin B12, and folate—all reported consequences of persistent *H. pylori* infection—are known to adversely affect cognitive and motor development in children [49, 50, 51]. Additionally, chronic low‐grade systemic inflammation induced by *H. pylori* may directly influence neurodevelopmental processes through inflammatory cytokine pathways [52, 53]. Emerging evidence also suggests that alterations in the gut microbiome and the microbiome–gut–brain axis caused by *H. pylori* infection could influence brain function and development [54]. These potential pathways underscore the complexity of mechanisms by which early‐life *H. pylori* infection may impact neurodevelopment.

Interestingly, the association between *H. pylori* infection and developmental delay in our study was most pronounced among children performing in the higher percentiles of developmental scores. This pattern may suggest that the impact of *H. pylori* infection is more pronounced in children with initially favorable developmental trajectories, potentially because these children have greater developmental potential at risk, whereas its effects could be less discernible among children already facing socioeconomic or health‐related challenges [55].

Strengths of this study include its prospective design, the use of a validated developmental screening tool appropriate for West African settings, and adjustment for key confounders. The application of quantile regression allowed us to assess differential effects across the distribution of developmental milestone scores, providing a more nuanced understanding of the association.

However, several limitations should be considered. The observational design precludes causal inference. Despite using a culturally adapted tool, subtle differences in milestone expression across contexts may influence results. Unmeasured confounding, such as micronutrient deficiencies, environmental enteropathy, or other undetected infections, cannot be excluded. While key confounders including socioeconomic status and sex were adjusted for, other factors such as hereditary influences, perinatal insults, and nutritional status were not examined and warrant further investigation in future studies. Finally, the relatively small number of *H. pylori* ‐positive children, while sufficient for detecting moderate associations, limits the power to detect smaller effects. The follow‐up time of 12 months does not permit drawing conclusions about quantitative and qualitative developmental deficits in the later stages of the children's development. However, it has been demonstrated, that pre‐ and postnatal harmful exposure, for example, to environmental toxins, has the potential to negatively affect long‐term neurocognitive development [56, 57].

While spontaneous clearance of *H. pylori* infection can occur in children, it is generally considered uncommon. Most infections, once established, tend to persist in the absence of treatment [58]. This supports the reliability of stool antigen testing to identify active infection relevant to neurodevelopmental outcomes in our cohort.

Child development in low‐income settings is shaped by a complex interplay of biological, environmental, and social factors including maternal feeding practices, childhood illnesses, nutritional status, and household environment. The association between *H. pylori* infection and motor development observed in our study should be interpreted within this multifactorial framework. These findings highlight the importance of considering infectious diseases alongside other determinants of child health when designing interventions to improve developmental outcomes.

Given the high prevalence of *H. pylori* infection in sub‐Saharan Africa and its early acquisition in infancy, the observed association with poorer developmental outcomes has important public health implications [48, 59]. Future longitudinal studies are needed to confirm these findings and clarify mechanisms, including direct inflammatory effects and those mediated by micronutrient deficiencies. Intervention studies evaluating the impact of early *H. pylori* prevention strategies or nutritional supplementation on developmental outcomes could provide critical insights [60].

Our results underscore the importance of integrating infection control strategies into early childhood development programs in low‐resource settings. While the effects of toxic exposures have been investigated systematically, further research on the intersection of infectious diseases and early brain development remains an urgent global health priority, especially where multiple biological and social adversities converge.

In conclusion, *H. pylori* infection at 12 months of age was independently associated with lower motor and total developmental milestone scores among children in Côte d'Ivoire and Ghana, particularly among those with higher developmental performance. These findings highlight a potentially underrecognized factor contributing to developmental disparities in resource‐limited settings and emphasize the need for further research on infection‐related neurodevelopmental risks in early childhood.

## Conflicts of Interest

The authors declare no conflicts of interest.

## Supporting information


**Table S1:** Univariable quantile regression model displaying associations between *H. pylori* status and developmental milestone scores in 12‐month‐old children at the 25th percentile, median and 75th percentile.
**Table S2:** Multivariable quantile regression model displaying associations between *H. pylori* status and developmental milestone scores in 12‐month‐old children at the 25th percentile, median and 75th percentile.
**Table S3:** Univariable quantile regression model displaying associations between maternal *H. pylori* status and developmental milestone scores in 12‐month‐old children at the 25th percentile, median and 75th percentile.
**Figure S1:** Developmental Milestones Checklist (DMC).

## Data Availability

The data that support the findings of this study are available on request from the corresponding author.
