# Supplementary material for: Helicobacter pylori and Impaired Early Childhood Development—Evidence From a Birth Cohort Study From Ghana and Côte d'Ivoire
Source: Helicobacter. 2025 Oct 31;30(5):e70087. doi: 10.1111/hel.70087 (PMC12578653; doi:10.1111/hel.70087)

**Supplementary Materials**

Supplementary Table S1: Univariable quantile regression model displaying associations between *H. pylori* status and developmental milestone scores in 12-month-old children at the 25th percentile, median and 75th percentile.

|  | Predictor Variable | Tau | Quantile Level | Estimate | Confidence Interval Lower Bound | Confidence Interval Upper Bound | P-value | FDR-corrected P-value |
| --- | --- | --- | --- | --- | --- | --- | --- | --- |
| Locomotor domain | *H. pylori* positive | 1 | 0.25 | -1 | -3.27 | 1.27 | 0.389 | 0.584 |
|  | *H. pylori* positive | 2 | 0.5 | 0 | -2.22 | 2.22 | 1.000 | 1.000 |
|  | *H. pylori* positive | 3 | 0.75 | -2 | -4.25 | 0.25 | 0.083 | 0.249 |
| Fine motor domain | *H. pylori* positive | 1 | 0.25 | 0 | -0.96 | 0.96 | 1.000 | 1.000 |
|  | *H. pylori* positive | 2 | 0.5 | 0 | -1.13 | 1.13 | 1.000 | 1.000 |
|  | *H. pylori* positive | 3 | 0.75 | -1 | -2.18 | 0.18 | 0.099 | 0.297 |
| Language domain | *H. pylori* positive | 1 | 0.25 | 0 | -3.77 | 3.77 | 1.00 | 1 |
|  | *H. pylori* positive | 2 | 0.5 | 1 | -3.18 | 5.18 | 0.64 | 1 |
|  | *H. pylori* positive | 3 | 0.75 | 0 | -4.46 | 4.46 | 1.00 | 1 |
| Personal-social domain | *H. pylori* positive | 1 | 0.25 | 1 | -0.40 | 2.40 | 0.163 | 0.351 |
|  | *H. pylori* positive | 2 | 0.5 | 0 | -1.74 | 1.74 | 1.000 | 1.000 |
|  | *H. pylori* positive | 3 | 0.75 | -1 | -2.64 | 0.64 | 0.234 | 0.351 |
| Total motor score | *H. pylori* positive | 1 | 0.25 | -1 | -3.99 | 1.99 | 0.512 | 0.512 |
|  | *H. pylori* positive | 2 | 0.5 | -2 | -5.54 | 1.54 | 0.269 | 0.404 |
|  | *H. pylori* positive | 3 | 0.75 | -5 | -8.49 | -1.51 | 0.005 | 0.015 |
| Total score | *H. pylori* positive | 1 | 0.25 | 1 | -5.40 | 7.40 | 0.760 | 0.760 |
|  | *H. pylori* positive | 2 | 0.5 | -3 | -10.75 | 4.75 | 0.449 | 0.673 |
|  | *H. pylori* positive | 3 | 0.75 | -10 | -18.04 | -1.96 | 0.016 | 0.048 |

Supplementary Table S2: Multivariable quantile regression model displaying associations between *H. pylori* status and developmental milestone scores in 12-month-old children at the 25th percentile, median and 75th percentile.

|  | Predictor Variable | Tau | Quantile Level | Estimate | Confidence Interval Lower Bound | Confidence Interval Upper Bound | P-value | FDR-corrected adjusted P-value |
| --- | --- | --- | --- | --- | --- | --- | --- | --- |
| Locomotor domain | *H. pylori* positive | 1 | 0.25 | -0.5 | -2.58 | 1.58 | 0.637 | 0.637 |
|  | Socioeconomic status high | 1 | 0.25 | -1.5 | -3.34 | 0.34 | 0.112 |  |
|  | Sex female | 1 | 0.25 | -0.5 | -2.34 | 1.34 | 0.595 |  |
|  | *H. pylori* positive | 2 | 0.5 | -1.0 | -3.43 | 1.43 | 0.421 | 0.631 |
|  | Socioeconomic status high | 2 | 0.5 | -1.0 | -3.03 | 1.03 | 0.336 |  |
|  | Sex female | 2 | 0.5 | -1.0 | -3.05 | 1.05 | 0.339 |  |
|  | *H. pylori* positive | 3 | 0.75 | -1.0 | -3.34 | 1.34 | 0.403 | 0.631 |
|  | Socioeconomic status high | 3 | 0.75 | -1.0 | -2.88 | 0.88 | 0.299 |  |
|  | Sex female | 3 | 0.75 | 0.0 | -1.91 | 1.91 | 1.000 |  |
| Fine motor domain | *H. pylori* positive | 1 | 0.25 | 0 | -0.99 | 0.99 | 1.000 | 1.000 |
|  | Socioeconomic status high | 1 | 0.25 | 0 | -0.86 | 0.86 | 1.000 |  |
|  | Sex female | 1 | 0.25 | 0 | -0.86 | 0.86 | 1.000 |  |
|  | *H. pylori* positive | 2 | 0.5 | 0 | -1.16 | 1.16 | 1.000 | 1.000 |
|  | Socioeconomic status high | 2 | 0.5 | 0 | -1.00 | 1.00 | 1.000 |  |
|  | Sex female | 2 | 0.5 | 0 | -1.01 | 1.01 | 1.000 |  |
|  | *H. pylori* positive | 3 | 0.75 | -2 | -3.26 | -0.74 | 0.002 | 0.006 |
|  | Socioeconomic status high | 3 | 0.75 | 1 | -0.56 | 2.56 | 0.210 |  |
|  | Sex female | 3 | 0.75 | 0 | -1.29 | 1.29 | 1.000 |  |
| Language domain | *H. pylori* positive | 1 | 0.25 | 1 | -2.66 | 4.66 | 0.593 | 1 |
|  | Socioeconomic status high | 1 | 0.25 | 1 | -1.87 | 3.87 | 0.495 |  |
|  | Sex female | 1 | 0.25 | 1 | -1.92 | 3.92 | 0.503 |  |
|  | *H. pylori* positive | 2 | 0.5 | 0 | -3.99 | 3.99 | 1.000 | 1 |
|  | Socioeconomic status high | 2 | 0.5 | 1 | -2.16 | 4.16 | 0.536 |  |
|  | Sex female | 2 | 0.5 | 2 | -1.15 | 5.15 | 0.215 |  |
|  | *H. pylori* positive | 3 | 0.75 | 0 | -4.70 | 4.70 | 1.000 | 1 |
|  | Socioeconomic status high | 3 | 0.75 | 4 | -0.24 | 8.24 | 0.066 |  |
|  | Sex female | 3 | 0.75 | 2 | -1.81 | 5.81 | 0.304 |  |
| Personal-social domain | *H. pylori* positive | 1 | 0.25 | 0 | -1.43 | 1.43 | 1.000 | 1.000 |
|  | Socioeconomic status high | 1 | 0.25 | 0 | -1.26 | 1.26 | 1.000 |  |
|  | Sex female | 1 | 0.25 | 1 | -0.25 | 2.25 | 0.117 |  |
|  | *H. pylori* positive | 2 | 0.5 | -1 | -2.61 | 0.61 | 0.226 | 0.678 |
|  | Socioeconomic status high | 2 | 0.5 | 0 | -1.44 | 1.44 | 1.000 |  |
|  | Sex female | 2 | 0.5 | 1 | -0.44 | 2.44 | 0.174 |  |
|  | *H. pylori* positive | 3 | 0.75 | 0 | -1.66 | 1.66 | 1.000 | 1.000 |
|  | Socioeconomic status high | 3 | 0.75 | 1 | -0.60 | 2.60 | 0.222 |  |
|  | Sex female | 3 | 0.75 | 0 | -1.55 | 1.55 | 1.000 |  |
| Total motor score | *H. pylori* positive | 1 | 0.25 | 0 | -3.00 | 3.00 | 1.000 | 1.000 |
|  | Socioeconomic status high | 1 | 0.25 | -1 | -3.78 | 1.78 | 0.482 |  |
|  | Sex female | 1 | 0.25 | 0 | -2.78 | 2.78 | 1.000 |  |
|  | *H. pylori* positive | 2 | 0.5 | -2 | -5.60 | 1.60 | 0.277 | 0.416 |
|  | Socioeconomic status high | 2 | 0.5 | -1 | -4.26 | 2.26 | 0.549 |  |
|  | Sex female | 2 | 0.5 | 0 | -3.29 | 3.29 | 1.000 |  |
|  | *H. pylori* positive | 3 | 0.75 | -7 | -11.06 | -2.94 | 0.001 | 0.003 |
|  | Socioeconomic status high | 3 | 0.75 | 5 | 0.35 | 9.65 | 0.036 |  |
|  | Sex female | 3 | 0.75 | 0 | -4.11 | 4.11 | 1.000 |  |
| Total score | *H. pylori* positive | 1 | 0.25 | 1.5 | -5.22 | 8.22 | 0.662 | 0.662 |
|  | Socioeconomic status high | 1 | 0.25 | -1.5 | -7.74 | 4.74 | 0.638 |  |
|  | Sex female | 1 | 0.25 | 1.5 | -4.79 | 7.79 | 0.641 |  |
|  | *H. pylori* positive | 2 | 0.5 | -3.0 | -10.71 | 4.71 | 0.446 | 0.662 |
|  | Socioeconomic status high | 2 | 0.5 | 1.0 | -6.18 | 8.18 | 0.785 |  |
|  | Sex female | 2 | 0.5 | 6.0 | -1.17 | 13.17 | 0.102 |  |
|  | *H. pylori* positive | 3 | 0.75 | -10.0 | -17.45 | -2.55 | 0.009 | 0.027 |
|  | Socioeconomic status high | 3 | 0.75 | 8.0 | 0.25 | 15.75 | 0.044 |  |
|  | Sex female | 3 | 0.75 | 5.0 | -2.49 | 12.49 | 0.192 |  |

Supplementary Table S3: Univariable quantile regression model displaying associations between maternal *H. pylori* status and developmental milestone scores in 12-month-old children at the 25th percentile, median and 75th percentile.

|  | Predictor Variable | Tau | Quantile Level | Estimate | Confidence Interval Lower Bound | Confidence Interval Upper Bound | P-value | FDR-corrected P-value |
| --- | --- | --- | --- | --- | --- | --- | --- | --- |
| Locomotor domain | Mother: *H. pylori* positive | 1 | 0.25 | -2 | -4.17 | 0.17 | 0.072 | 0.216 |
|  | Mother: *H. pylori* positive | 2 | 0.5 | -1 | -3.22 | 1.22 | 0.379 | 0.379 |
|  | Mother: *H. pylori* positive | 3 | 0.75 | -1 | -3.17 | 1.17 | 0.367 | 0.379 |
| Fine motor domain | Mother: *H. pylori* positive | 1 | 0.25 | 0 | -1.03 | 1.03 | 1.000 | 1 |
|  | Mother: *H. pylori* positive | 2 | 0.5 | 0 | -1.18 | 1.18 | 1.000 | 1 |
|  | Mother: *H. pylori* positive | 3 | 0.75 | -1 | -3.07 | 1.07 | 0.346 | 1 |
| Language domain | Mother: *H. pylori* positive | 1 | 0.25 | -1 | -4.49 | 2.49 | 0.575 | 0.637 |
|  | Mother: *H. pylori* positive | 2 | 0.5 | -1 | -4.92 | 2.92 | 0.618 | 0.637 |
|  | Mother: *H. pylori* positive | 3 | 0.75 | 1 | -3.15 | 5.15 | 0.637 | 0.637 |
| Personal-social domain | Mother: *H. pylori* positive | 1 | 0.25 | -1 | -2.42 | 0.42 | 0.168 | 0.364 |
|  | Mother: *H. pylori* positive | 2 | 0.5 | 0 | -1.60 | 1.60 | 1.000 | 1.000 |
|  | Mother: *H. pylori* positive | 3 | 0.75 | 1 | -0.67 | 2.67 | 0.243 | 0.364 |
| Total motor score | Mother: *H. pylori* positive | 1 | 0.25 | -1 | -4.43 | 2.43 | 0.568 | 0.643 |
|  | Mother: *H. pylori* positive | 2 | 0.5 | -3 | -6.84 | 0.84 | 0.128 | 0.384 |
|  | Mother: *H. pylori* positive | 3 | 0.75 | -1 | -5.22 | 3.22 | 0.643 | 0.643 |
| Total score | Mother: *H. pylori* positive | 1 | 0.25 | -9.88 | 3.88 | 0.394 | 0.591 | 0.760 |
|  | Mother: *H. pylori* positive | 2 | 0.5 | -12.71 | 2.71 | 0.205 | 0.591 | 0.673 |
|  | Mother: *H. pylori* positive | 3 | 0.75 | -7.65 | 9.65 | 0.821 | 0.821 | 0.048 |

Supplementary Figure S1: Developmental Milestones Checklist (DMC).


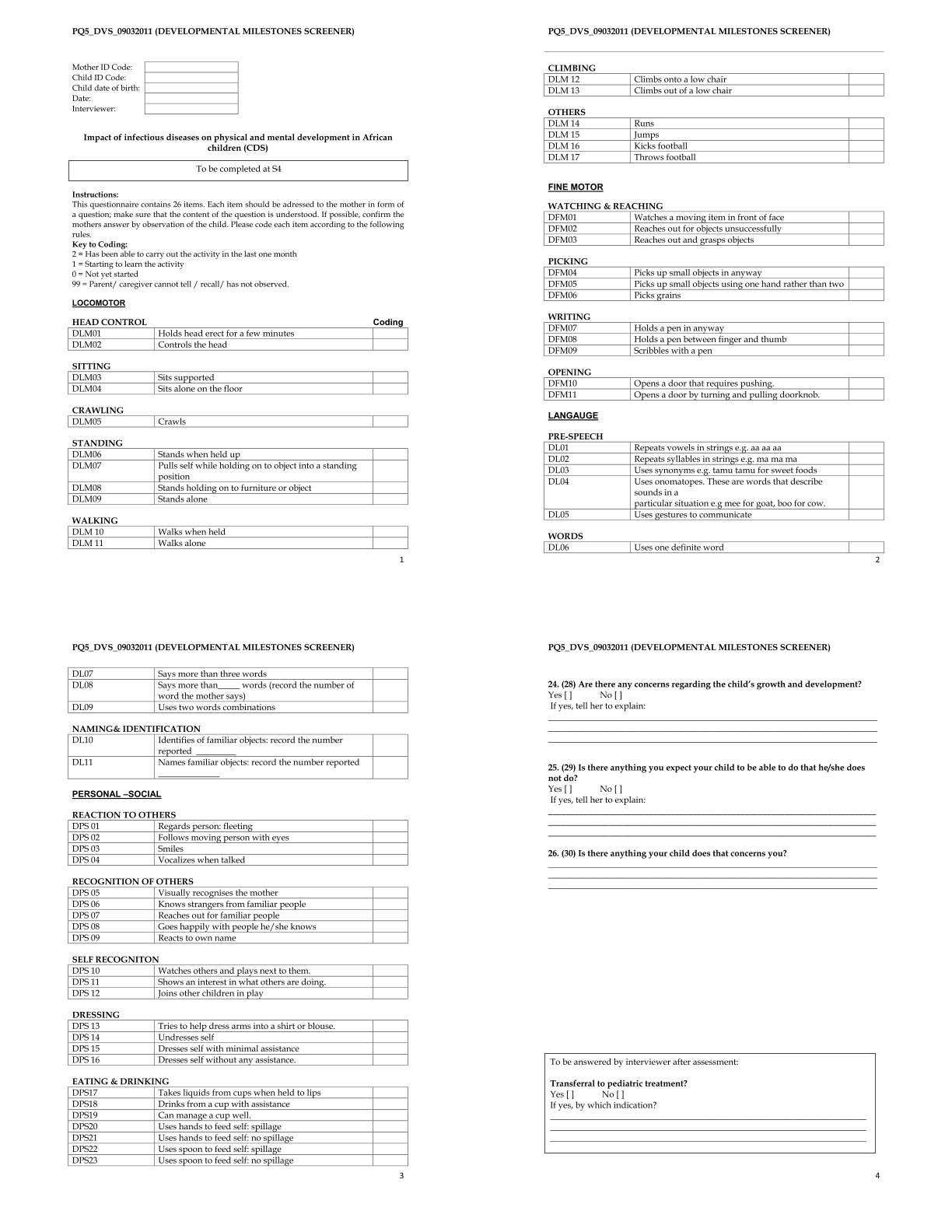

Supplement: Supplementary file 1 — Table S1: Univariable quantile regression model displaying associations between H. pylori status and developmental milestone scores in 12‐month‐old children at the 25th percentile, median and 75th percentile. Table S2: Multivariable quantile regression model displaying associations between H. pylori status and developmental milestone scores in 12‐month‐old children at the 25th percentile, median and 75th percentile. Table S3: Univariable quantile regression model displaying associations between maternal H. pylori status and developmental milestone scores in 12‐month‐old children at the 25th percentile, median and 75th percentile. Figure S1: Developmental Milestones Checklist (DMC). [file HEL-30-e70087-s001.docx]
